# LncRNA-HAGLR motivates triple negative breast cancer progression by regulation of WNT2 via sponging miR-335-3p

**DOI:** 10.18632/aging.203272

**Published:** 2021-08-10

**Authors:** Liting Jin, Chenggang Luo, Xinhong Wu, Manxiu Li, Shun Wu, Yaojun Feng

**Affiliations:** 1 Department of Breast Surgery, Hubei Cancer Hospital, Tongji Medical College, Huazhong University of Science and Technology, Wuhan City, Hubei Province 430079, China; 2 Department of Radiology, Hubei Cancer Hospital, Tongji Medical College, Huazhong University of Science and Technology, Wuhan City, Hubei Province 430079, China

**Keywords:** triple negative breast cancer, lncRNA HAGLR, miR-335-3p, WNT2, tumor progression

## Abstract

Background: Triple negative breast cancer (TNBC) is a group of highly heterogeneous mixed breast cancer at the level of gene expression profile. Therefore, it is of great clinical significance to explore the molecular mechanism of TNBC and find a targeted therapeutic approach from the molecular level.

Methods: Long non-coding RNA (lncRNA) HAGLR expression level was measured by and qRT-PCR in TNBC tissues and cell lines. EdU, MTT, wound healing and Transwell assays were performed to explore the role of HAGLR on the malignancy of TNBC cells. Luciferase assay was used to clarify the binding between miR-335-3p with HAGLR and WNT2. The tumor formation experiment in nude mice was used to explore the function of HAGLR *in vivo*.

Results: HAGLR was increased in TNBC tissues and cell lines. Silencing of HAGLR inhibited viability, proliferation, migration, and invasion of BT549 cells. Furthermore, HAGLR acted as a sponge of miR-335-3p and inhibited its expression. And miR-335-3p directly targeted WNT2. Functionally, forced expression of miR-335-3p or knockdown of WNT2 removed the promoted effects of lncRNA HAGLR on TNBC development. *In vivo* tumorigenesis experiments indicated HAGLR accelerated tumor growth via miR-335-3p/WNT2 axis.

Conclusion: Our study revealed that HAGLR promoted the growth of TNBC, which was mediated by miR-335-3p/WNT2 axis.

## INTRODUCTION

Triple negative breast cancer (TNBC) is a subtype of breast cancer, which has negative expression of the estrogen receptor, progesterone receptor and human epidermal growth factor receptor 2 expression [1, 2]. And TNBC accounts for 10% ~ 20% of all breast cancer pathology types [3]. The most common type of TNBC is invasive ductal carcinoma, followed by the metaplastic carcinoma [4]. TNBC mainly occurs in premenopausal young women, which is characterized by high degree of malignancy and poor prognosis. 30% ~ 40% of TNBC can be developed of into metastatic breast cancer, especially the lungs and brain metastasis. TNBC recurrence rate is higher than other subtypes within five years after diagnosis, but fell rapidly after five years. The recurrence rate after eight years even is lower than other breast cancer subtypes [5]. With the innovation of precision medical era gene detection technology, TNBC has been found to be a group of highly heterogeneous mixed breast cancer at the level of gene expression profile. Thus, it is an inevitable trend to explore new methods of TNBC treatment at the gene level.

In 1961, people first came into contact with the concept of messenger RNA (mRNA). With the gradual establishment of the rule of genetic center, mRNA, which acts as a template for protein translation, is accepted by the public as the transmitter of genetic information. When the human genome was sequenced, 98.5% of the genome was transcribed to long non-coding RNAs (lncRNAs) whose length were more than 200nt [6]. Since lncRNAs do not contain an open reading frame and cannot encode proteins, lncRNAs are considered as metabolic wastes. It was not until 2002 that Okazaki et al. [7] first discovered that lncRNAs can be used as important regulatory RNAs, which can regulate genes, interfere with transcription, and regulate protein functions. LncRNAs are also involved in cell proliferation and differentiation, as well as embryonic growth and development.

As we all know, the occurrence of cancer is mainly caused by abnormal gene expression. A large number of lncRNAs have been found to be abnormally expressed in various cancers by regulating gene expression [8]. LncRNAs may play a role in epigenetic changes as oncogenes and/or tumor suppressors. LncRNA HLA complex P5 (HCP5) was induced in TNBC cell lines and tumor tissues [9]. Down-regulation of HCP5 can promote apoptosis and inhibit tumor growth. MiR-219a-5p is reported to be a miRNA that inhibits a variety of cancers [10]. The level of miR-219a-5p was down-regulated in TNBC cells, while forced expression of miR-219a-5p interfered HCP5 and BIRC3. Overexpression of HCP5 decreased miR-219a-5p, but increased BIRC3 mRNA levels. HCP5 positively correlated with BIRC3 expression, activating BIRC3 in the apoptotic signaling pathway by competing with miR-219a-5p and promoting TNBC development [11]. LncRNA HAGLR was shown to be involved in the process of various cancers. HAGLR was increased in esophageal cancer and acted as a sponge of miR-143-5p, which promoted EMT (epithelial-mesenchymal transition) associated genes expression and migration of esophageal cancer cells [12]. As well, HAGLR promoted the development of non-small cell lung cancer [13]. However, whether HAGLR contributes to TNBC remains undiscovered.

Herein, we aimed to explore the effect of lncRNA HAGLR in TNBC, and further illuminate the possible underlying mechanisms.

## RESULTS

### Up-regulation of lncRNA HAGLR in TNBC tissues and cells

**Figure 1:**
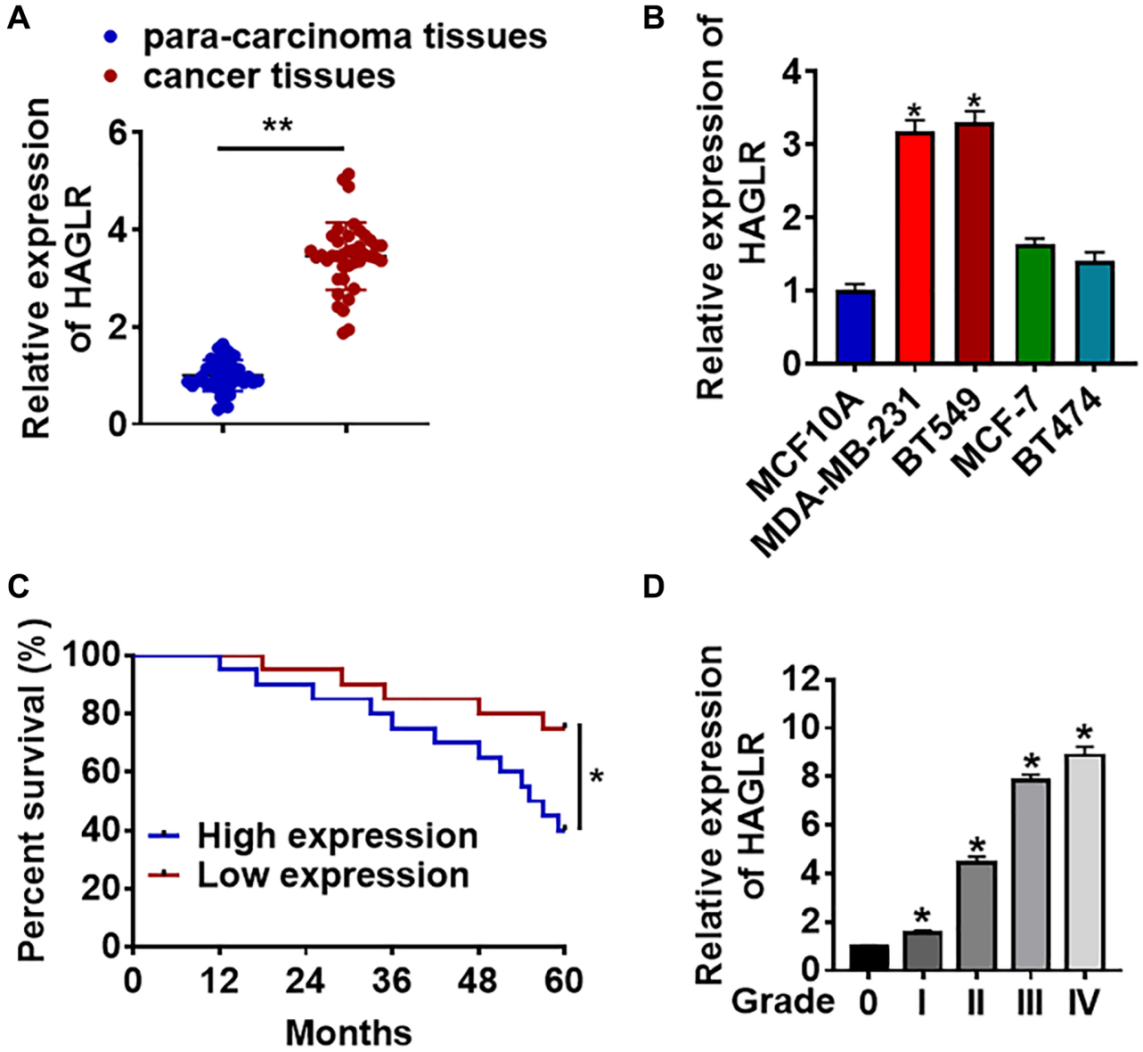
**The expression of lncRNA HAGLR in TNBC tissues and cells.** We collected 40 samples of patients diagnosed with TNBC. (**A**) The expression of HAGLR in para-carcinoma and cancer tissues was detected by qRT-PCR. (**B**) qRT-PCR analysis for HAGLR level in normal breast cell MCF10A and TNBC cell lines MDA-MB-231 and BT549. (**C**) According to the mean level of HAGLR in Figure 1A, 40 TNBC patients was divided into low (*n* = 20) and high expression group (*n* = 20). Kaplan-Meier curves indicated 5-year survival rate of TNBC patients. (**D**) Another 40 TNBC patients were collected, which includes grade 0 to grade IV of TNBC (*n* = 8 for each grade), and qRT-PCR was used to test HAGLR level in different grades. Data are mean ± SD; ^*^P < 0.05, ^**^P < 0.01.

In 40 samples of patients diagnosed with TNBC, lncRNA HAGLR was highly expressed in TNBC cancer compared with para-carcinoma tissues (Figure 1A). In addition, we cultured TNBC cell lines (BT549 and MDA-MB-231) and normal breast cell MCF10A, and found the level of HAGLR was dramatically higher in TNBC cell lines than that in normal breast cell (Figure 1B). In addition, HAGLR was also up-regulated in non TNBC cell lines (MCF-7 and BT474), but the increase of HAGLR in non-TNBC cells was lower than that in TNBC cells (Figure 1B). According to the mean level of HAGLR in Figure 1A, 40 TNBC patients were divided into low (*n* = 20) and high expression group (*n* = 20). Kaplan-Meier curves indicated 5-year survival rate of TNBC patients was significantly higher in low expression patients than high expression patients (Figure 1C). Furthermore, we collected TNBC tissues from different grades (grade 0 to grade IV, *n* = 8) of TNBC, and found a positive correlation between HAGLR and tumor grades (Figure 1D). Together, these data indicated that HAGLR might be involved in TNBC progression.

### Silencing lncRNA HAGLR inhibited malignancy of TNBC cells

**Figure 2:**
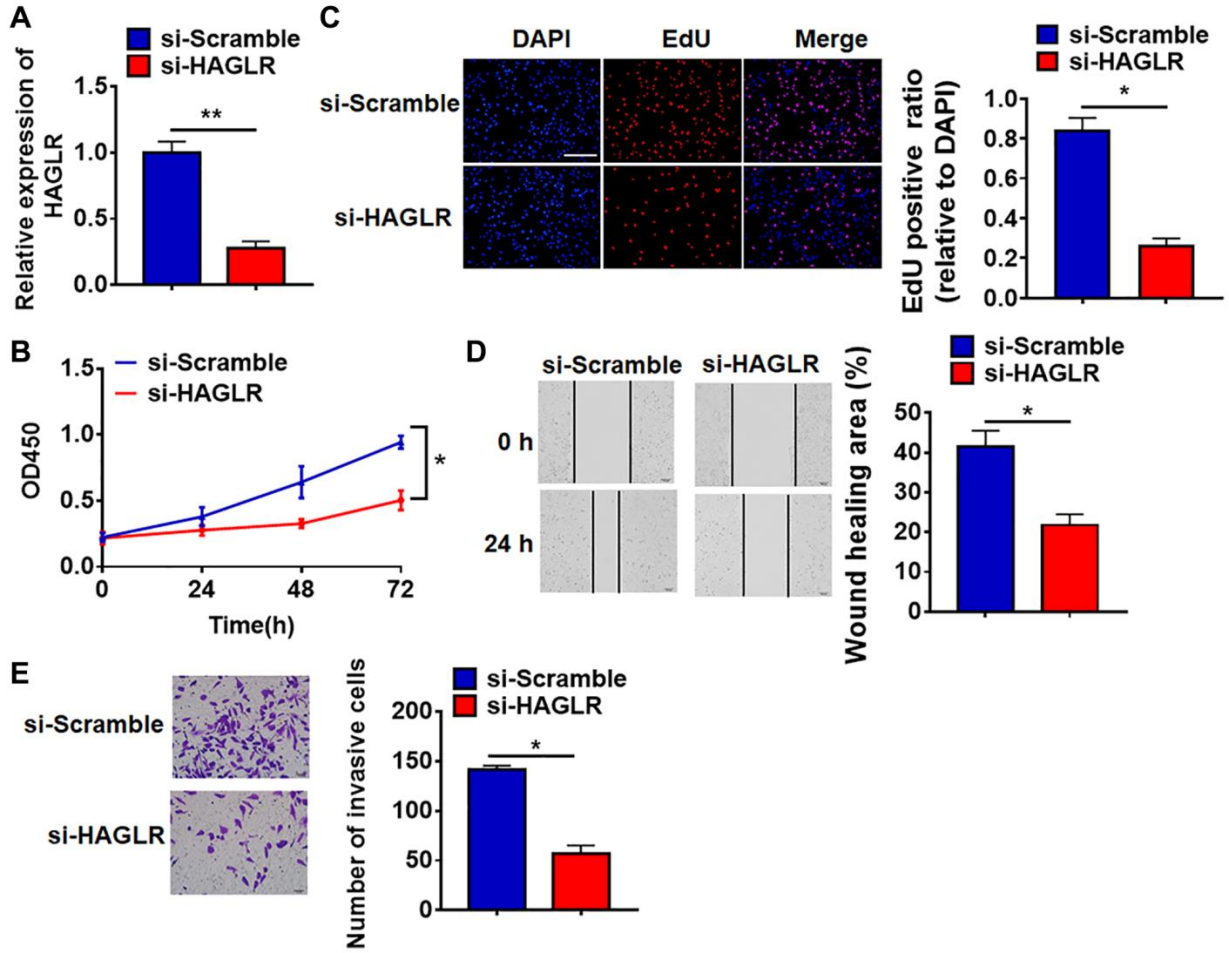
**Deletion of HAGLR inhibited the viability, proliferation, migration and invasion of BT549 cells.** Small interfering RNA of HAGLR (si-HAGLR) and its Scramble were transfected into BT549 cells. (**A**) The knockdown efficiency of si- HAGLR was determined by qRT-PCR. (**B**) CCK8 assay was used to test viability of BT549 cells. (**C**) EdU assay was to detected proliferation of BT549 cells. Scale bar, 100 μm. (**D**) Wound healing assay was to evaluate migration of BT549 cells. Scale bar, 100 μm. (**E**) Transwell assay was to examine invasion of BT549 cells. Scale bar, 50 μm. Data are mean ± SD; ^*^P < 0.05, ^**^P < 0.01.

To further identify the function of HAGLR in TNBC progression, we constructed small interfering RNA of HAGLR (si-HAGLR). As showed in Figure 2A, transfection of si-HAGLR significantly suppressed the expression of HAGLR in BT549 cells comparing to scramble siRNA (si-Scramble). CCK-8 experiment was performed to detect the viability in BT549 after HAGLR siRNAs transfection. The results showed that loss of HAGLR significantly inhibited growth rate of BT549 cells at 48 h and 72 h than cells transfected with scrambled siRNA (Figure 2B). EdU analysis was performed to detect cell proliferation, which showed that deletion of HAGLR decreased EdU positive cell numbers (Figure 2C). Then, wound healing suggested silencing HAGLR decreased the wound healing area, which exhibited a lower migratory ability in si-HAGLR transfected cells (Figure 2D). Transwell assay showed that si-HAGLR reduced the invasive cell ability in BT549 cells (Figure 2E). These results indicated that knockdown of HAGLR suppressed TNBC malignancy.

### HAGLR suppressed miR-335-3p expression

**Figure 3:**
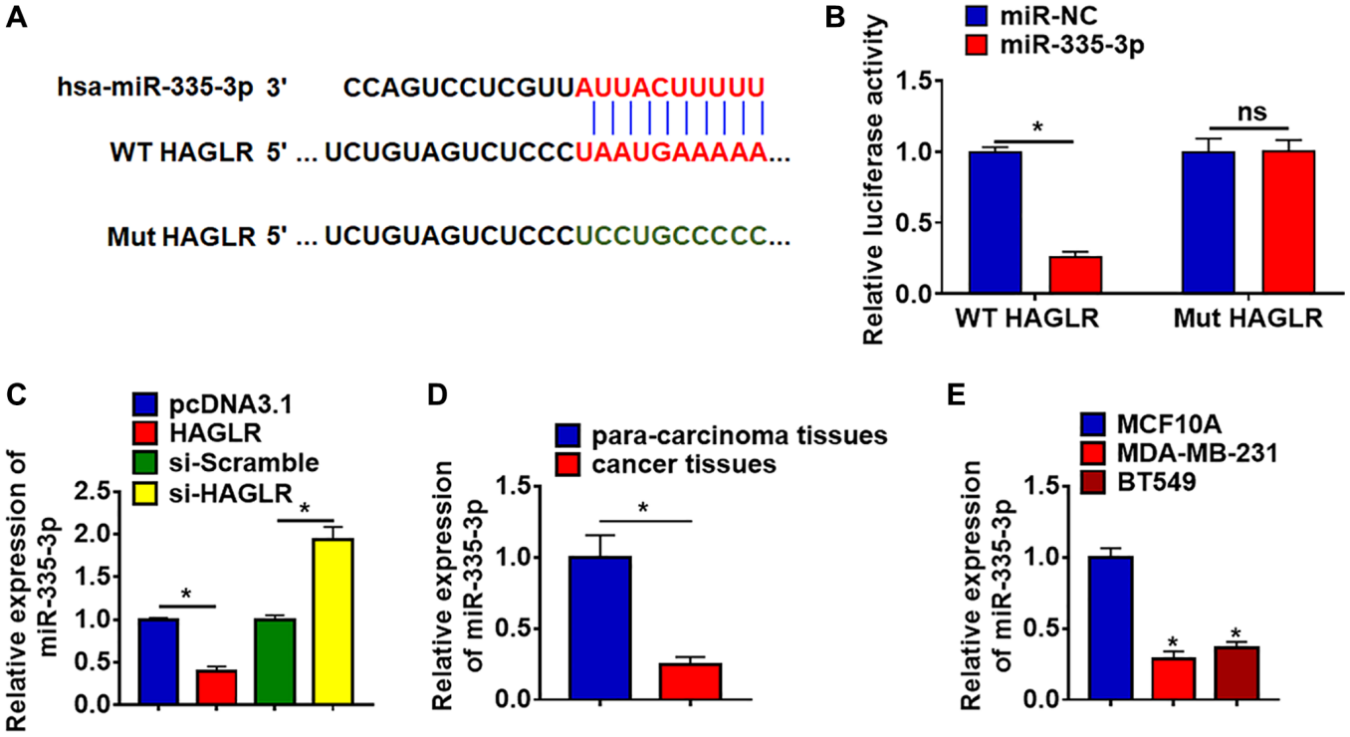
**HAGLR acted as a sponge of miR-335-3p and inhibited its expression.** (**A**) MiRanda database showing the binding sites of miR-335-3p with HAGLR, and the mutant sequence of miR-335-3p. (**B**) Wild type and mutant miR-335-3p was transfected into HEK293 cells with or without HAGLR, and luciferase assay was to evaluate the binding between miR-335-3p and HAGLR. (**C**) BT549 cells were transfected with HAGLR plasmid or si-HAGLR or its NC, the mRNA level of miR-335-3p was detected using qRT-PCR. (**D**) The expression of miR-335-3p in para-carcinoma and cancer tissues was detected by qRT-PCR. (**E**) qRT-PCR analysis for miR-335-3p level in normal breast cell MCF10A and TNBC cell lines MDA-MB-231 and BT549. Data are mean ± SD; ^*^P < 0.05, Abbreviation: ns: no statistical significance.

To clarify the underlying mechanism of HAGLR in TNBC regulation, we used miRanda database to identify miRNA with HAGLR biding sites, which showed a binding between miR-335-3p and HAGLR (Figure 3A). Luciferase assay showed that HAGLR inhibited luciferase activity of WT miR-335-3p, but not mutant miR-335-3p (Figure 3B). Furthermore, qRT-PCR analysis showed overexpression of HAGLR significantly inhibited miR-335-3p level, while si-HAGLR promoted miR-335-3p expression (Figure 3C). In addition, the mRNA level of miR-335-3p was significantly decreased in TNBC tissue and cells (Figure 3D and 3E).

### MiR-335-3p directly targeted WNT2

**Figure 4:**
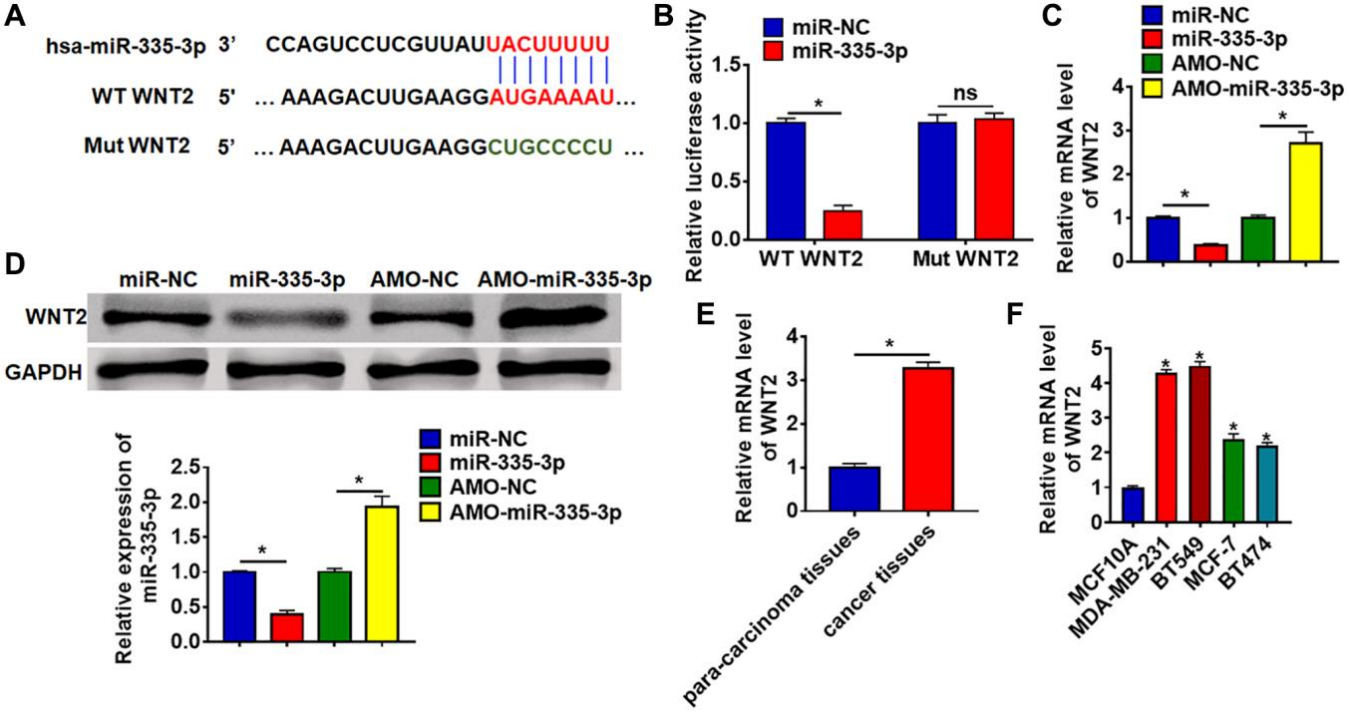
**WNT2 was a directed target of miR-335-3p.** (**A**) The binding bases of miR-335-3p and WNT2 from Targetscan. (**B**) Wild type and mutant WNT2 was transfected into HEK293 cells with or without miR-335-3p, and luciferase assay was to evaluate the binding. BT549 cells were transfected with miR-335-3p or AMO-miR-335-3p, (**C**) the mRNA level and (**D**) the protein level of WNT2 was detected. (**E**–**F**) The expression of WNT2 in TNBC tissues and cells was determined by qRT-PCR. Data are mean ± SD; ^*^P < 0.05, Abbreviation: ns: no statistical significance.

The data from Targetscan (http://www.targetscan.org/vert_72/) showed that the 3′UTR of WNT2 possessed a direct target site for miR-335-3p (Figure 4A). And luciferase activity of WT WNT2, but not mutant WNT2, was decreased in the miR-335-3p mimic group compared with NC group (Figure 4B). Furthermore, miR-335-3p significantly inhibited both the mRNA and protein expression of WNT2, while AMO-335-3p increased WNT2 level in TNBC cells (Figure 4C and 4D). What’s more, we detected WNT2 expression in clinical TNBC tissues and cells, which showed a dramatic increase of WNT2 level in TNBC tissues and cells (Figure 4E and 4F).

### HAGLR promoted growth via miR-335-3p/WNT2 axis in TNBC cells

**Figure 5:**
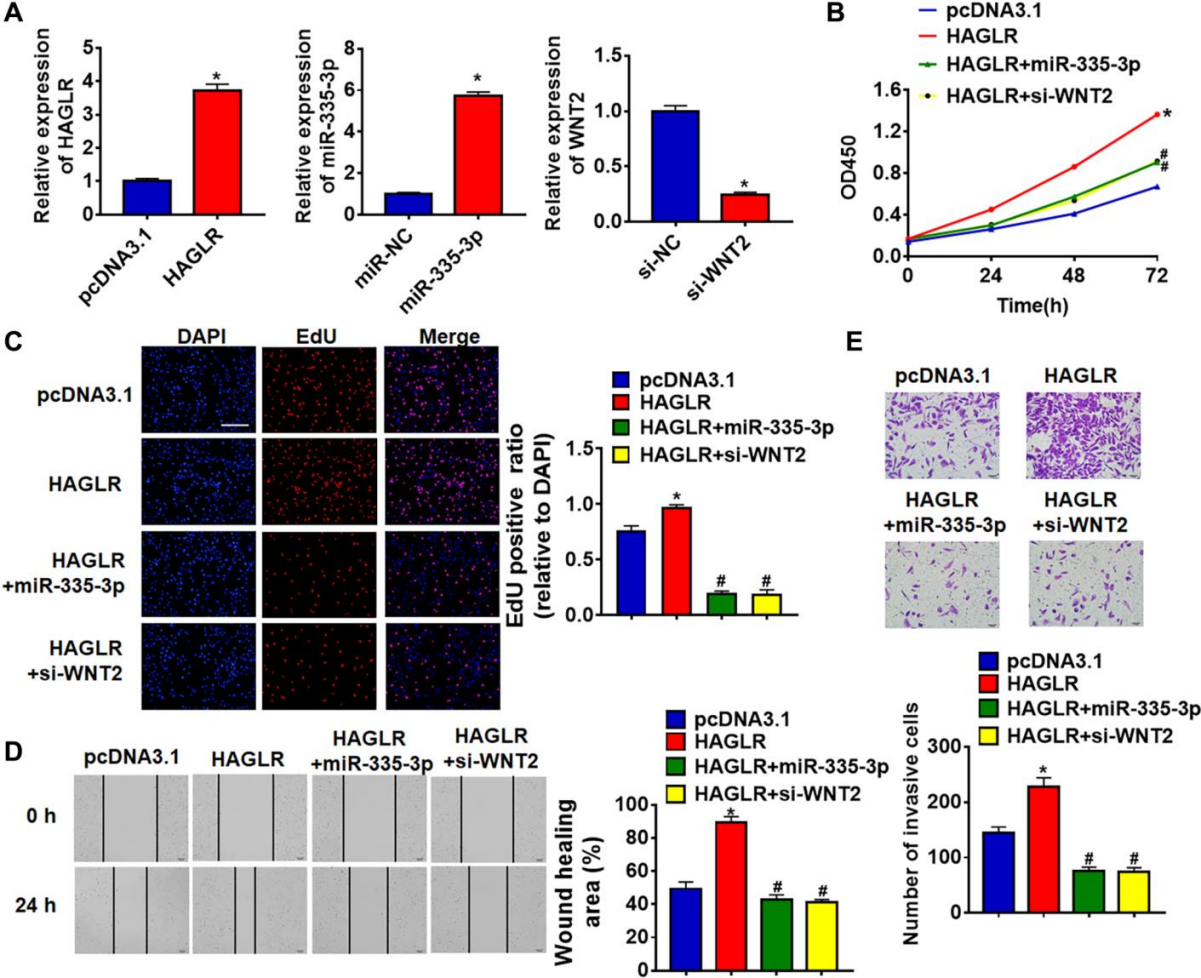
**HAGLR promoted TNBC growth through miR-335-3p/WNT2 axis.** HAGLR was transfected into BT549 cells with miR-335-3p or si-WNT2. (**A**) The transfection efficiency was detected using qRT-PCR. (**B**) CCK8 assay for cell viability of BT549 cells. (**C**) EdU assay for cell proliferation of BT549 cells. Scale bar, 100 μm. (**D**) Wound healing assay for cell migration of BT549 cells. Scale bar, 100 μm. (**E**) Transwell assay for cell invasion of BT549 cells. Scale bar, 50 μm. Data are mean ± SD; ^*^P < 0.05 vs pcDNA3.1, ^#^*P* < 0.05 vs HAGLR.

We then forced expression of HAGLR in BT549 cells (Figure 5A). HAGLR accelerated cell viability and promoted cell viability, proliferation, migration and invasion in BT549 cells (Figure 5B–5E). Additionally, overexpression of miR-335-3p or loss of WNT2 reversed the promoting effect of HAGLR on TNBC cells (Figure 5B–5E).

### HAGLR promoted TNBC tumorigenesis *in vivo*

**Figure 6:**
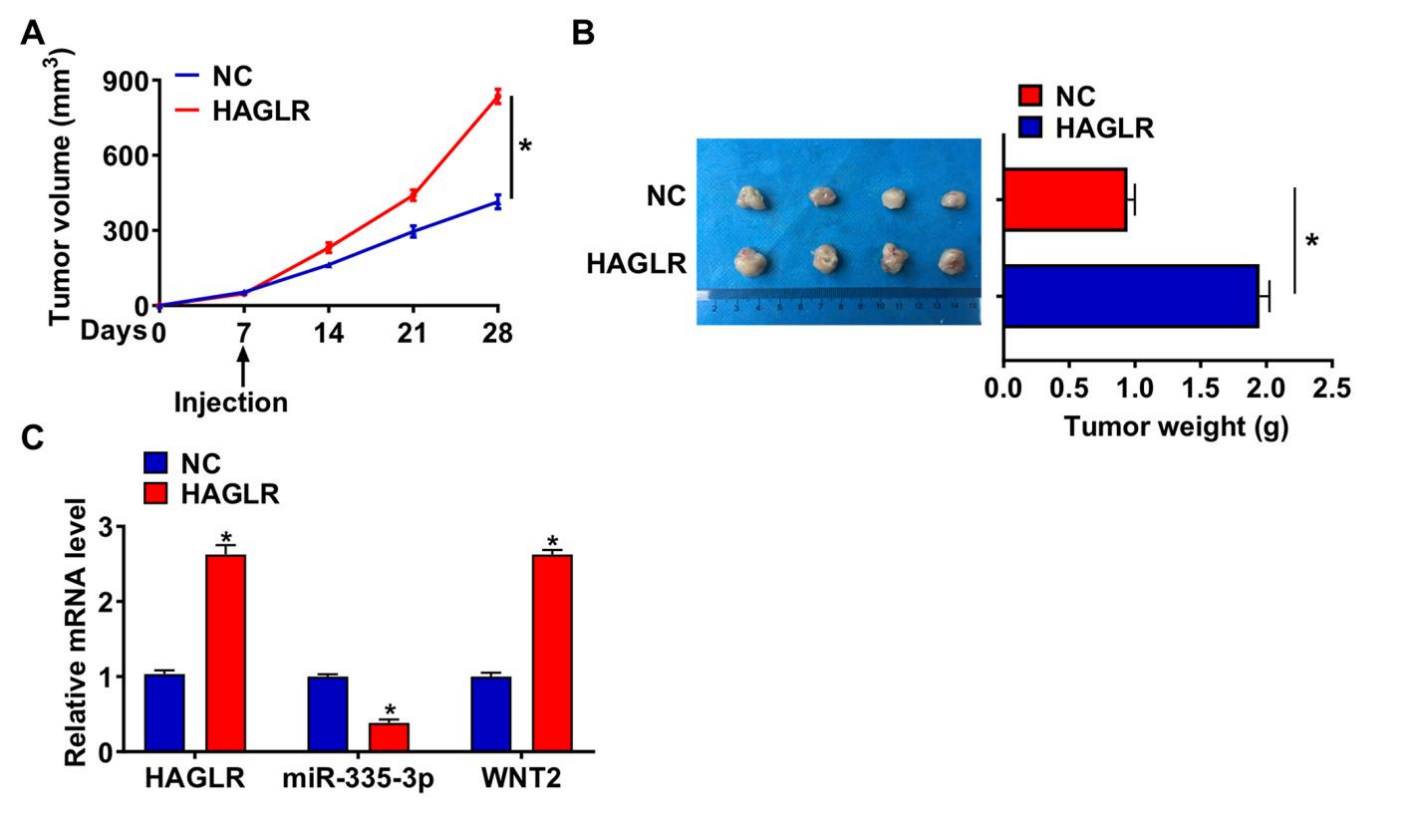
**HAGLR promoted TNBC growth *in vivo*.** 30 mice were divided into two group randomly, BT549 cells was subcutaneously injected into nude mice. 1 week later, we injected lentivirus packaged HAGLR or NC into tumors. (**A**) Tumor volume was measured every 7 days. (**B**) Tumors was isolated after 28 days of BT549 cells injection, and photos for representative tumors. (**C**) The mRNA of HAGLR, miR-335-3p, and WNT2 in isolated tumors were detected by qRT-PCR. Data are mean ± SD; ^*^P < 0.05.

To further explore the function of HAGLR in TNBC, we set up a xenograft nude mice model. 12 mice were divided into two groups randomly, BT549 cells were subcutaneously injected into nude mice. One week later, we injected lentivirus packaged HAGLR into tumors, and we measured tumor volume. The mice with HAGLR showed a bigger tumor volume, and tumors grew faster (Figure 6A). The tumors were isolated 28 days after injection, and HAGLR significantly increased tumor weight (Figure 6B). In addition, isolated these tumor tissues had a higher HAGLR level after injection of lentivirus packaged HAGLR (Figure 6C). Moreover, injection of HAGLR decreased the mRNA level of miR-335-3p increased WNT2 expression (Figure 6C). Taken together, HAGLR regulated the progression of TNBC via miR-335-3p /WNT2 axis.

## DISCUSSION

TNBC has a high short-term recurrence rate after surgery, and is prone to visceral metastasis, especially lung and brain metastasis [14]. In addition, the chemotherapy was not effective for late TNBC [15]. Compared with other subtypes of breast cancer, TNBC has a high degree of malignancy and poor prognosis [16], which has been the focus and difficulty of clinical research.

Numerous studies have revealed that lncRNAs contribute to the development of various cancers, acting as a tumor promoter or suppressor [17]. High expression of lncRNA HOTAIR promoted the metastasis of lung cancer cells to normal tissues, which was mainly achieved through the recruitment of PRC2. And a higher level of HOTAIR is related to a poor prognosis of lung cancer [18]. Compared with the adjacent normal tissue samples, lncRNA TUSC7 in gastric cancer tissues is significantly lower. And increasing the expression of TUSC7 suppressed tumor growth [19]. HAGLR is a novel lncRNA mainly studied in lung cancer [20] and serous ovarian carcinoma [21]. In the present study, we found that HAGLR was up-regulated in TNBC tissues and cell lines compared to normal tissue and cell lines. To further identify the role of HAGLR in TNBC, we constructed siRNA for HAGLR to inhibit its expression in BT549 cells. Followed functional analysis showed that silencing of HAGLR inhibited growth and migration of BT549 cells.

It has been reported that lncRNAs can act as a competitive endogenous RNA (ceRNA) to inhibit microRNAs (miRNAs) function. MiRNAs inhibit the transcription and translation of mRNAs essential for tumor development [22]. In hepatocellular carcinoma, the binding of HULC to miR-372 attenuates miRNA-mediated translation inhibition of PRKACB and induces the phosphorylation of CREB [23]. Thus, we used miRanda database to identify miRNA with HAGLR biding sites, which showed a binding between miR-335-3p and HAGLR. And Targetscan database showed that WNT2 might be a direct target of miR-335-3p. Then luciferase assay indicated that HAGLR acted as a sponge of miR-335-3p and inhibited its expression. WNT2 served as a downstream molecule of miR-335-3p in TNBC.

Wnt is a secreted glycoprotein, which can be over-activated when the cell change’s external factors lead to dysregulation, abnormal body development, and even tumor formation [24]. The Wnt signaling pathway contributes to the proliferation, growth, and metastasis of various malignancies [25]. Wnt2 is a critical molecule in the Wnt signaling pathway. Studies have shown that a large amount of Wnt2 secretion can activate the wnt2-expanded catenin-protein signaling pathway and ultimately promote the growth of cancer cells [26]. Moreover, Wnt2 expression is positively correlated with the invasion potential, tumor stage, and clinical grade of malignant tumors [27]. In the present study, we found the forced expression of miR-335-3p or knockdown of WNT2 removed the promoted effects of lncRNA HAGLR on TNBC development. *In vivo* tumorigenesis experiments indicated HAGLR accelerated tumor growth via miR-335-3p/WNT2 axis.

Many cancer-related lncRNAs regulate specific targets of different tumor types through a variety of mechanisms. Although there has been some understanding of lncRNAs, there are still many problems to be solved, including how to screen lncRNAs with abnormal tumor-related expression early and how to intervene lncRNAs to improve tumor treatment. In tumor tissues, abnormal expression and mutation of lncRNA are specific, which can generally predict the prognosis of patients.

## CONCLUSIONS

In conclusion, our study revealed lncRNA HAGLR promoted the development of TNBC by upregulating WNT2 via sponging miR-335-3p. Our study might provide a novel target for clinical prevention and treatment of TNBC.

## MATERIALS AND METHODS

### Clinical samples

Fresh cancer tissue samples and para-carcinoma tissue samples were taken from 40 TNBC patients undergoing surgical procedures at Hubei Cancer Hospital (Table 1). All of the patients or their guardians provided written consent. The studies involving human participants were reviewed and approved by Ethics Committee of Hubei Cancer Hospital.

**Table 1:** Clinical characteristics of TNBC patients.

**Characteristics**	***n* (40)**	**Percentage (%)**
**Age**		
≤50	**25**	**62.50**
>50	**15**	**37.50**
**Tumor stage**		
I-II stage	**12**	**30.00**
III-IV stage	**28**	**70.00**
**Tumor size**		
≤5 cm	**14**	**35.00**
>5 cm	**26**	**65.00**
**Lymphatic metastasis**		
Positive	**8**	**20.00**
Negative	**32**	**80.00**
**Distant metastasis**		
M0	**30**	**75.00**
M1	**10**	**25.00**

### Cell culture and treatment

The cell lines were purchased from the Science Cell Laboratory. Cells were cultured in RPMI 1640 (GIBCO, USA) supplemented with 10% fetal bovine serum (Cromwell, USA) and 100 μL/mL penicillin and streptomycin (Sigma-Aldrich, USA) and placed at 37°C with 5% CO_2_. To confirm the role of HAGLR on TNBC, 2000 ng HAGLR plasmid or small interfering RNA (siRNA) of HAGLR (si-HAGLR) was transfected into BT549 cells using Lipo3000 (Invitrogen, Carlsbad, CA, USA). 50 nmol miR-335-3p mimics or 2000 ng si-WNT2 was transfected into BT549 cells. The plasmid or miRNA mimics or antisense oligodeoxynucleotide of miR-9-3p (AMO-335-3p) or small interfering RNA (si-RNA) or negative control (NC) were constructed and purchased from by Ribobio company (Guangzhou, China). The sequence of siRNAs: si-HAGLR: 5′-UUAGAAGAGGGAUAACAUCAG-3′, si-WNT2: 5′-UUUAUGGAGUUGUCAAAGCUG-3′.

### RNA isolation and qRT-PCR

RNA isolation, reverse transcription and quantitative expression were carried according to manufacturer’s instructions. All the kits were purchased from Vazyme, and gene expression was calculated using 2^−ΔΔCt^ method. Primer list: HAGLR (F: 5′-GGGCTGGTACAGACTAGGGA-3′, R: 5′-TAAGCAGGTCAGAAAGGGCG-3′), GADPH (F: 5′-AACGGATTTGGTCGTATTG-3′, R: 5′-GGAAGATGGTGATGGGATT-3′), miR-335-3p (F: 5′-TTTTTCATTATTGCTC-3′, R: 5′-GTGCAGGGTCCGAGGT-3′), U6 (F: 5′-CTCGCTTCGGCAGCACA-3′, R: 5′-ACGCTTCACGAATTTTGCGT-3′).

### Protein isolation and Western blot

Tissues and cells were lysed with RIPA lysis Mix. Western blotting assay was performed as previously described. Briefly, 60 μg protein extractions were loaded via SDS-PAGE and transferred onto nitrocellulose membranes (absin, China), then incubated with primary antibodies for 2 h at room temperature, then plated at 4°C overnight. The membranes were incubated in 5% non-fat milk blocking buffer for horizontal mode 3 h. After incubation with secondary antibodies, the membranes were scanned using an Odyssey, and data were analyzed with Odyssey software (LI-COR, USA). Primary antibodies list: WNT 2 (27214-1-AP, Proteintech) and GADPH (12935-1-AP, Proteintech).

### CCK8 assay

BT549 cells were seeded in 96-well cell plates and added CCK-8 solution (Vazyme, China) at 0, 24, 48, and 72 h. 2 h later, measure the OD value at 450 nm.

### EdU assay

BT549 cells were inoculated in a 24-well plate. 100 μL EdU (50 μM) solution (Ribobio, China) was added into cells 24 hours later, and incubated in the cell incubator for 2 hours. The medium was discarded, and the cells were washed with PBS for 3 times. The cells were fixed with 4% paraformaldehyde at room temperature for 30 min and washed 3 times with PBS. The cells were incubated with 0.5% Triton-100 for 10 min and washed with PBS for 3 times. DAPI was used to stain the nuclei for 5 min. After washing with PBS, the cells were observed under a microscope and photographed.

### Wound healing assay

BT549 cells (5 × 10^5^) were planted in a 6-well plate, and when the cells grew to fuse, two vertical parallel lines were drawn with 10 μL suction head against the ruler. The floating cells were washed with PBS and cultured in a serum-free medium for 24 h. Images were taken at 0 and 24 h of cell culture, respectively.

### Transwell assay

BT549 cells in the logarithmic growth phase were adjusted to 2 × 10^5^ cells/well of medium (without serum) and plated into the upper chamber insert pre-coated with 1 μg/μl Matrigel. The lower chamber was added with 500 μL of medium (with 10% FBS), and then incubate the chamber at 37°C for 48 h. Then the invading cells were visualized by the crystal violet and inverted microscope.

### Luciferase assay

The wildtype (WT) 3′-UTR of HAGLR or WNT2 conserved miR-335-3p binding sites and mutated (Mut) 3′-UTRs were synthesized by Invitrogen. The fragment was subcloned into the SacI and HindIII sites downstream of the luciferase gene in the pMIR Reporter. MiR-NC or miR-335-3p mimics was transfected with luciferase reporter vectors into HEK293 cells. The cells were collected after 48 h post-transfection and lysed to detected the luciferase activity (Promega).

### *In vivo* tumor growth assay

BT549 cells (5 × 10^6^) were subcutaneously injected in the right lower limb of the nude mice (*n* = 6 per group). 1 week later, we injected lentivirus packaged HAGLR or negative control (NC) (constructed by Shanghai Genechem Co., LTD.) into tumors, and we measured tumor volume every 7 days. After 28 days of injection, mice were intraperitoneally injected with 3% pentobarbital sodium. They were killed by anesthesia with CO_2_, and the tumors were removed for follow-up study. This study was reviewed and approved by Ethics Committee of Hubei Cancer Hospital. The animal experiments involved in this study were carried out following the Guidelines for the Care and Use of Laboratory Animals issued by the National Institutes of Health (NIH).

### Statistical analysis

All data is presented as a mean ± SD. Statistical analysis was performed using Student’UTRs *t*-test or Wilcoxon test or a one-way ANOVA through Graphpad 7.0 and SPSS 22.0.
